# Time-Aware Dual LSTM Neural Network with Similarity Graph Learning for Remote Sensing Service Recommendation

**DOI:** 10.3390/s24041185

**Published:** 2024-02-11

**Authors:** Jinkai Zhang, Wenming Ma, En Zhang, Xuchen Xia

**Affiliations:** School of Computer and Control Engineering, Yantai University, Yantai 264005, China; zhangjinkai@s.ytu.edu.cn (J.Z.); zhangen0522@s.ytu.edu.cn (E.Z.); 202100358072@s.ytu.edu.cn (X.X.)

**Keywords:** remote sensing resource service, history behavior sequences, similarity relationship graph structure, long short-term memory, graph convolutional networks

## Abstract

Technological progress has led to significant advancements in Earth observation and satellite systems. However, some services associated with remote sensing face issues related to timeliness and relevance, which affect the application of remote sensing resources in various fields and disciplines. The challenge now is to help end-users make precise decisions and recommendations for relevant resources that meet the demands of their specific domains from the vast array of remote sensing resources available. In this study, we propose a remote sensing resource service recommendation model that incorporates a time-aware dual LSTM neural network with similarity graph learning. We further use the stream push technology to enhance the model. We first construct interaction history behavior sequences based on users’ resource search history. Then, we establish a category similarity relationship graph structure based on the cosine similarity matrix between remote sensing resource categories. Next, we use LSTM to represent historical sequences and Graph Convolutional Networks (GCN) to represent graph structures. We construct similarity relationship sequences by combining historical sequences to explore exact similarity relationships using LSTM. We embed user IDs to model users’ unique characteristics. By implementing three modeling approaches, we can achieve precise recommendations for remote sensing services. Finally, we conduct experiments to evaluate our methods using three datasets, and the experimental results show that our method outperforms the state-of-the-art algorithms.

## 1. Introduction

Recently, remote sensing technologies have made remarkable progress. Constant innovations in this field have led to advancements in sensor technology, multi-modal data processing, and geographic information analysis. Comprehending the Earth’s surface and its transformations has driven research and has allowed global challenges to be tackled in areas such as Earth science, environmental science, and meteorology. Remote sensing technology is a method of observing the Earth’s surface from a distance. It allows scientists to gather extensive data with high resolutions, both spatially and temporally. This technology is widely used in the military sector and national security for tasks like border monitoring, reconnaissance, and surveillance of hazardous areas. It provides an abundance of experimental data and intelligence support.

The demand for remote sensing data has increased significantly in various fields. Researchers from different domains strongly support the high precision and extensive coverage of remote sensing data, which significantly enhance the effectiveness of research methodologies. As a result, a substantial volume of remote sensing resources has been generated. However, users find it challenging to fully comprehend and locate the resources they need within the system. Even if they are aware of the remote sensing resources available, they still struggle to find exactly what they need for a specific task.

The remote sensing service systems are designed to integrate information from various sources. Each type of remote sensing resource in the system is specialized, requiring users to have a considerable level of expertise in their respective knowledge domains. The system is open and provides resources to users free of charge. However, due to the complexity and diversity of resources in the system, users find it challenging to explore the relevant resources accurately. Even with substantial experience, users struggle to pinpoint their specific needs, leading to an impractical search process.

Most current remote sensing information service systems rely on keyword search and subscription modes to provide users with the required resources [1]. In the search mode, users can enter specific query conditions and conduct searches accordingly. The system will return relevant resources in terms of the search conditions. In the subscription mode, users can submit their requirements to the service as resource orders. This mode can proactively push recommendations to users when data that meet their requirements become available. While these two modes precisely cater to user needs and generate accurate recommendations based on specific requirements, they also present particular challenges. The user must be very familiar with the domain knowledge. This can make it challenging for users to get started and potentially decrease their satisfaction. It can be challenging for experienced users to package their requirements effectively. These modes primarily rely on a simple resource filtering approach, limiting users’ potential to explore other resources and broaden their expertise. Additionally, the modes obscure the similarity between remote sensing resources, making it difficult for users to discover resources similar to their needs.

There is a growing preference among current users for a system that generates comprehensive and timely recommendations by taking into account their historical needs. Researchers have proposed various solutions to address these issues. Analysis of user preferences has resulted in precise recommendations for remote sensing resources, thereby enhancing the user experience. In the context of remote sensing service recommendations, this refers to the application of recommendation systems in remote sensing resources. Machine learning models or deep learning models, among others, are utilized to analyze the historical behavioral patterns of users when using remote sensing resource information service systems to determine users’ behavioral preferences more accurately. Subsequently, this model was deployed as a web service on a server to achieve recommendations for remote sensing resources.

Several researchers have tackled information filtering issues in remote sensing information service systems using conventional collaborative filtering (CF) techniques [2,3,4,5,6,7]. However, traditional CF methods can only reveal superficial linear relationships and cannot capture complex nonlinear relationships due to their inherent structure. Furthermore, this approach overlooks the contextual information present in the system, failing to explore other information on recommendation performance.

As a result, some researchers have been exploring the significance of contextual information and its role in service recommendations [8,9,10,11,12,13]. By incorporating more contextual information, researchers can gain a better understanding of the impact of user interactions on recommendation outcomes. However, it has been observed that researchers often fail to consider the effect of nonlinear relationships on recommendation performance when integrating contextual information. The emergence of deep learning has enabled researchers to explore complex and nonlinear relationships [14,15,16,17,18,19]. This has led to significant improvements in recommendation performance, particularly in remote sensing information service recommendations and other similar domains.

However, while deep learning can explore intricate nonlinear relationships, it may not be optimal for data represented as graph structures. To address this, some special neural networks have been invented, such as Graph Neural Networks (GNNs) and others, which are designed to handle graph-structured data. By perceiving the positional structural information of nodes within a graph and comprehending relationships between nodes through information propagation mechanisms, GNNs can enable more sophisticated exploration. Researchers have therefore incorporated Graph Neural Networks into service recommendations to achieve better recommendation outcomes [20,21,22,23]. Although service recommendation methods have achieved good results, they still have some shortcomings. For instance, some approaches overlook the importance of initializing node embeddings in graph-structured data, which can mask critical information during aggregation through Graph Neural Networks, such as the similarity between nodes. Additionally, certain methods fail to consider the temporal dynamics of users’ historical behaviors when making service recommendations, thus neglecting the influence of time sequences on users. Finally, some methods do not take into account users’ latent factors, which can prevent the provision of a flexible vector encoding to learn the unique properties of these latent influences.

In this study, we propose using a Time-aware Dual LSTM Neural Network with Similarity Graph Learning to address specific issues related to the remote sensing service recommendation (TGDL-RSSR). The method comprises three parts, which are designed to identify users’ unique latent influences, the temporal dynamics of their historical behaviors, and the similarity and graph structure characteristics among different remote sensing information categories. First, we organize user interactions in a time-ordered sequence based on their past behavior in the remote sensing service system. A relationship graph is created based on the connections between different remote-sensing resource categories. Then, we use collaborative filtering to construct a cosine similarity matrix that measures the similarity between category relationships. Second, we utilize an embedding layer to represent the historical interaction sequences of users. We use LSTM to capture the temporal dynamics and establish relationships between users’ next search results and their historical behavior. We use a Multi-Layer Perceptron (MLP) layer based on the cosine similarity matrix to create embedded representations of remote sensing information that share similar characteristics. We utilize GCN to model the graph structure data and this embedding, allowing us to capture the similarity and adjacency relationships among categories. We use a second LSTM to model the temporal dynamics among similarities by combining the user’s historical sequence. Using an embedding layer, we identify the user’s unique ID. Finally, after obtaining three embeddings, we concatenate them and use an MLP to predict the remote sensing resource category for users.

The main contributions of this study are summarized as follows:

- We propose a method that utilizes LSTM to capture the dynamic relationships between search items and construct a sequence of user search history behavior.
- We present a method that utilizes CF to create a similarity matrix between remote sensing resource categories and then uses an MLP to generate embedded representations based on the matrix.
- We utilize GCN to model both the graph structure and similarity embeddings by establishing an adjacency graph relationship among categories.
- We utilize LSTM to capture the temporal dynamical similarity between categories by combining historical behavior sequences with GCN modeling results.

## 2. Related Work

Remote sensing is a scientific and technological discipline that uses sensors installed in satellites to collect the surface information of the Earth. Amidst the ongoing advancements in satellite technology and radar sensors, remote sensing has made remarkable progress, resulting in better data acquisition rates, accuracy, and resolution of Earth’s surface resources. The deployment of high-resolution, multispectral, and synthetic aperture radar satellites has significantly improved remote sensing in areas like land cover, resource monitoring, and environmental protection [24].

The use of Light Detection and Ranging (LiDAR) technology is becoming increasingly common in areas like digital terrain modeling, urban planning, and forestry. This technology provides highly accurate three-dimensional surface information [25]. Remote sensing technology plays a crucial role in monitoring climate change, assessing natural disasters, and generating useful data for environmental protection and climate research [26]. The integration of optical sensors and remote sensing technology has further expanded applications in environmental monitoring, agriculture, and urban planning. For detailed information on the remote sensing methods, refer to Table 1. Integrating remote sensing data with graphical information systems enables applications in urban planning, resource management, and geographic information science [27].

Remote sensing data have various characteristics, including diversity, multiple sources, temporal variability [28,29,30], and an increasing spatial resolution due to the advancement in sensing technologies [31]. These extensive high-resolution datasets provide more rich geographical information, allowing for a more thorough exploration of geographic features. This, in turn, facilitates comprehensive studies of the Earth system and the discovery of more intricate relationships [32].

Remote sensing data are widely applied in various fields, such as environmental monitoring and protection, urban planning and management, and water resources management. As remote sensing technology has developed rapidly, the amount of data collected has become increasingly massive, and the relationships within the data have become more complex, making it more challenging to analyze and research the data effectively. To address this challenge, remote sensing active service technology has emerged. This technology enables the analysis and exploration of vast remote sensing data, uncovering inherent patterns and subsequently generating a supportive service model [1,33].

Remote sensing technology has the potential to assist researchers in finding the right remote sensing data. However, traditional active service approaches rely on catalog searches and resource downloads, which require researchers to have a high level of proficiency in stating their needs. This approach may limit users’ perspectives and fail to suggest similar resources, making it hard to discover new remote sensing services. The timely updating of remote sensing data makes it challenging to generate accurate recommendations based on the timeliness of remote sensing resources and the users’ behavior [34]. Poor recommendations can significantly affect the user experience and the efficient utilization of remote sensing resources. For traditional active service techniques, in addition to catalog searching, other methods include surveys, interviews, archival research, and on-site investigations. These methods primarily conduct searches based on the analysis and positioning of user-defined needs. Similar to catalog searching, they face a common challenge of being unable to generate more precise recommendations for users based on their historical behavior. Detailed information is provided in Table 2.

Researchers have proposed solutions to address the limitations of traditional remote sensing active service techniques. One such solution is the FIR method, which was introduced by Lu et al. [35]. This method recommends and ranks remote sensing images based on users’ specific areas of interest by employing two special features. These features reflect correlations between users’ interests and remote-sensing images and serve as indicators of the relationship between them. Fuzzy association rule mining is then carried out based on these features to identify the relationship between the features and user interests. Additionally, two fuzzy inference strategies have been introduced to make recommendations in terms of the discovered association rules.

Hong et al. introduced a cluster-based index structure that is specifically designed for managing a large number of remote sensing images [36]. They proposed two indicators to measure the scalability and centrality between users’ area interests and remote-sensing resources.

Zhang et al. provided a method called the Spatio-Temporal Periodic Task model (STPT) [37] for recommending remote sensing data. This approach uses a probabilistic latent topic model to represent user retrieval behavior as a mixture of latent tasks. Two distributions are introduced to capture the relationships between tasks and spatial, temporal, and image features. Finally, an inference algorithm is employed to achieve the remote sensing service recommendation.

Li et al. proposed a technique to handle adaptive remote sensing recommendations based on behavioral analysis [38]. This method involves the real-time collection of user activities on the platform to discover their historical and dynamic preferences. Subsequently, it integrates this information using a decay method, ultimately achieving high-performance recommendations.

Song et al.provided a method to recommend remote-sensing resources using CF [39]. This method introduces a noise reduction technique in the similarity calculation process to determine the similarity between remote sensing data. The technique stabilizes the rating curve, which improves the recommendation performance.

Chu et al. introduced a new personalized remote-sensing images recommendation framework [40]. Their  method employs a knowledge graph to model the relationships between different entities. Using this information, this framework describes the connections between remote sensing images and users. The approach uses a Multi-attribute Fusion-based CF Network that utilizes the nonlinear computational capabilities of deep learning to calculate scores for each candidate image. This enables the recommendation of remote-sensing images that are tailored to individual users.

Li et al. provided a solution to the challenges of the rapid increase in remote sensing data [41]. In their method, they first utilized YOLOv3 object detection to extract the position distribution vectors of targets in remote sensing images as content information. Secondly, they constructed a multi-element user interest profile, which was dynamically adjusted according to the user’s active search records to enhance the recommendation performance. Finally, they achieved accurate and intelligent recommendations by matching image content, attributes, and user profile models.

Wang et al. proposed a novel Multi-modal Knowledge Graph-aware Deep Graph Attention Network (MMKDGAT) constructed based on graph convolutional networks [42]. This approach uses remote sensing resource images to create a multi-modal knowledge graph and incorporates additional information. Subsequently, a deep Graph Attention Network (GAT) is employed to mine information from this graph structure, aiming to capture information within various multi-modal nodes better and achieve more accurate recommendations.

Some of the current methods for analyzing remote sensing incorporate diversities and temporal characteristics. Some of these methods use knowledge graphs to incorporate this information. However, these approaches do not consider the temporal regularities or time dynamics of user behavior. As a result, they do not provide a comprehensive analysis of users’ long and short-term interests, which is crucial for identifying user preferences and behavior patterns, uncovering behavior patterns, understanding interest trends, and providing dynamic recommendations of remote sensing data that change over time. Although some research considers the similarity between remote sensing resources, the computation of this similarity often remains limited to linear operations like CF. This neglects deeper information about the similarity and the graph structure relationships among remote sensing resources.

Double LSTM refers to combining two LSTM models to explore sequential data further; each LSTM has distinct functionalities. Many researchers have applied double LSTMs in various fields. For example, Long et al. used double LSTMs to predict the trajectories of surrounding vehicles [43]. In this method, the driver’s historical sequential trajectories are input into the first LSTM to identify the driver’s intentions. Then, the output of the first LSTM, along with the driver’s historical geographic location sequence, is input into the second LSTM to predict future trajectories. Combining these two LSTMs allows a deeper exploration of the driver’s historical behavior, leading to more accurate predictions. Shi et al. proposed using double LSTMs to predict the Remaining Useful Life (RUL) of sensors [44]. The two LSTMs are responsible for monitoring changes in sensor data and predicting RUL, respectively. This combination allows for better representation of long- and short-term dependencies within the sensor data, resulting in more accurate predictions. Although these methods differ from ours in application domains, they combine two LSTMs to unearth higher-order information hidden in users’ historical behavior sequences.

To address the issues summarized, we propose a framework called Time-aware Dual LSTM Neural Network with Similarity Graph Learning for recommending remote sensing services (TGDL-RSSR). This framework constructs a historical behavior sequence based on the user’s interactions with remote sensing sources. It then uses LSTM to explore the user’s long- and short-term interests. Subsequently, CF is used to create a similarity matrix by considering the co-occurrence of remote-sensing resource categories. The MLP’s nonlinear capability is utilized to capture the relationships between similarities, achieving a category representation with deep-level similarities. Using obtained category representations and GCN, we simultaneously build an interaction graph structure to explore the adjacency relationships between remote sensing resource categories. Utilizing users’ historical behavior sequences, updated representations, and LSTM, we capture the potential temporal dynamics of these categories. Furthermore, we use the unique identifier ID of the user to explore their potential interests. This helps us to implement a remote sensing resource recommendation model. With this approach, the remote sensing resource service platform can analyze the temporal interests of users, similarities among remote sensing resources, and more.

## 3. Proposed Method

Within this section, we first formulate the problem and explain the relevant symbols used in this study. Then, we provide an overall introduction to our approach. Subsequently, we introduce each part of the model, which includes the input layer, representation layer, connection layer, multilayer perceptron layer, output layer, and optimization function.

### 3.1. Problem Formulation

**Figure 1:**
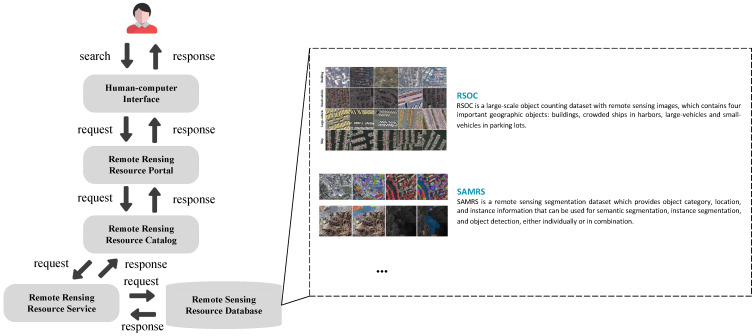
Typical remote sensing service system.

Remote sensing resource service systems typically consist of various components, such as human–computer interaction, a resource portal, a category directory, resource services, and databases [1]. The system’s workflow is illustrated in Figure 1, and it primarily functions by filtering resources using users’ search keywords, generating relevant results, and presenting them to users. However, this keyword-based search approach is not perfect, since it requires users to encapsulate their needs accurately. Users’ professional knowledge and ability affect the accuracy of the search conditions. If users fail to provide accurate search keywords, this can result in inaccurate search conditions and, consequently, incorrect search results.

As a result of this precise query approach, the system often filters resource categories similarly to users’ search keywords, leading to overly narrow search results. This search method does not analyze users’ past behavior, so the presented results may not be representative, requiring users to sift through a large number of search results to find what they need. This process significantly diminishes the user experience. Moreover, filtering out similar remote sensing resource categories limits users’ possibilities to explore other similar categories and narrows their perspective, which may not be ideal in some cases.

### 3.2. Definitions

**Definition** **1** (User and Remote sensing resources)**.** *U and V represent the set of users and remote sensing resources, respectively. $u_{i}$ is used to denote a user, and $v_{j}$ is used to denote a remote sensing resources, where $u_{i}\in U,v_{j}\in V$.*

**Definition** **2** (User unique identification ID)**.** *Let $I^{u_{i}}$ represent the ID of a specific user.*

**Definition** **3** (users’ historical behavior sequence)**.** *Let {$v_{t_{1}}^{u_{i}},v_{t_{2}}^{u_{i}},v_{t_{3}}^{u_{i}},...,v_{t_{m}}^{u_{i}}$} represent the users’ historical behavior sequence. In the sequence, t represents time, $t_{i}$ denotes the i-th time, and $v_{t_{i}}^{u_{i}}$ signifies the interaction between a user and a resource at time $t_{i}$. Here, $t_{i}<t_{i+1}$, i<m−1, m is the length of the sequence.*

**Definition** **4** (Similarity matrix of remote sensing resource categories)**.** *Let $A^{s}$ represent the similarity matrix among remote sensing resource categories. Where $A^{s}\in R^{\left|V|\times |V\right|}$, |V| represents the total number of remote sensing resource categories, $A_{v_{i}v_{j}}^{s}$ represents the similarity between category $v_{i}$ and category $v_{j}$.*

**Definition** **5** (Adjacency graph structure of remote sensing resource categories)**.** *Let $A^{I}$ represent the adjacency graph structure among remote sensing resource categories, where $A^{I}\in R^{\left|V|\times |V\right|}$, $A_{v_{i}v_{j}}^{I}$ represents the adjacency relationship between category $v_{i}$ and category $v_{j}$.*

The meanings of important notations used in this paper are shown in Table 3.

### 3.3. Overall Framework

**Figure 2:**
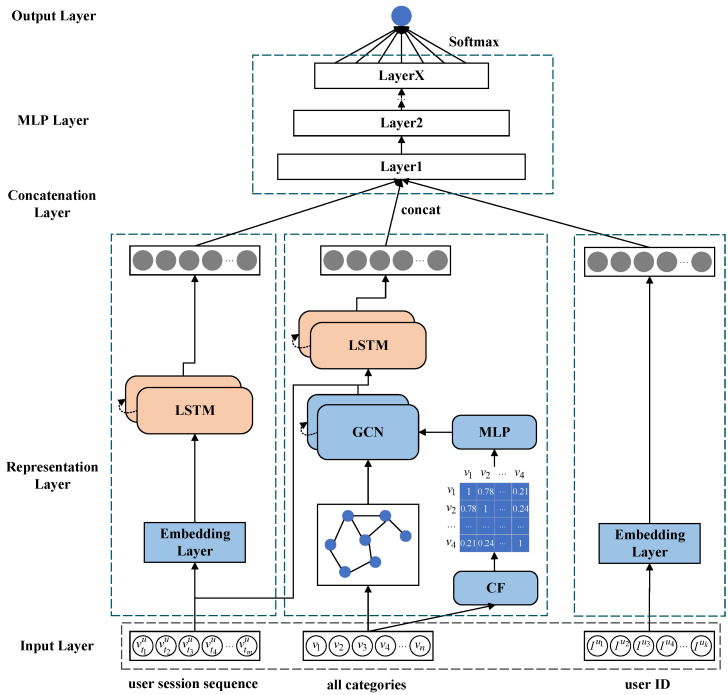
The overall architecture of TGDL-RSSR.

We propose a novel neural network framework to address current issues in remote sensing information service systems with time awareness and similarity graph learning, which is illustrated in Figure 2. This framework includes five modules: the input layer, representation layer, connection layer, MLP layer, and output layer. The core part of this model is the representation layer, which comprises three main modules.

The first module captures the historical behavior sequences of users when searching for remote sensing resources. It analyzes the long- and short-term interests in historical patterns for each user, which is crucial for accurately predicting their future needs.

The second module discovers the similarity and adjacency relationships among remote sensing resource categories in the system. We model the cosine similarity matrix through CF and MLP to obtain feature representations containing similarity between categories. We construct an interaction graph based on the adjacency relationships between categories and use GCN to model features and graph structures, thereby uncovering the similarity relationships among categories. We then combine the features aggregated by users’ historical behavior sequences and GCN, using LSTM to model them and extract the temporal regularities of category similarity. This is important for recommending resources similar to the user’s current needs.

The third module uses an embedding layer to model the user’s unique identification ID. This is used to uncover the user’s potential and unique factors when searching for resources, as each user has a distinctive behavior consciousness.

### 3.4. Input Layer

The input layer plays a crucial role in defining the model’s inputs. As the model’s core part is divided into three modules, the input layer is also categorized into three types.

The first type is the search history behavior sequence of users. This sequence is denoted as $H_{1}=\left\{v_{t_{1}}^{U},v_{t_{2}}^{U},v_{t_{3}}^{U},\cdots ,v_{t_{m}}^{U}\right\}$ and represents the user’s search history behavior. Here, $H_{1}\in R^{L\times M}$, and *L* is the sequence length, and *M* represents the number of user search interactions. For this sequence, the elements are arranged chronologically, with each element representing the user’s historical search behavior at a specific moment. In other words, each element corresponds to the category of remote sensing resources the user searched for at that particular time.

The second type is the set of all categories to which remote sensing resources belong in the system. Denoted as $H_{2}=\left\{v_{1},v_{2},v_{3},\ldots ,v_{n}\right\}$, where $H_{2}\in R^{\left|V\right|}$ and n=|V|. Each category is assigned a numerical identifier for extracting similarity matrices and constructing interaction graphs.

The third type of input is the set of all user IDs with a certain amount of historical interactions in the system. Denoted as $H_{3}=\left\{I^{u_{1}},I^{u_{2}},I^{u_{3}},\ldots ,I^{u_{k}}\right\}$, where $H_{3}\in R^{\left|U\right|}$ and k=|U|.

### 3.5. Representation Layer

The representation layer is responsible for obtaining critical feature representations required by the model. This layer comprises three primary modules: (1) Long Short-Term Interest Representation of Users’ Historical Behavior Sequences, (2) Time-dynamic Representation of Similarity Relationships among Remote Sensing Resource Categories, and (3) Representation of Potential Influencing Factors for User Unique Identification ID. Each representation method will be explained in detail below.

#### 3.5.1. Long Short-Term Interest Representation of Users’ Historical Behavior Sequences

The input for this module corresponds to the first type of input, which is denoted as $H_{1}$ in Section 3.4. In this type of input, each element in the sequence is essentially an index representation of a remote sensing resource category, with an additional characteristic of time. To process this input, we first flatten all user behavior sequences, resulting in a flattened shape of $H_{1}^{\prime }\in R^{L\times M}$. Subsequently, we apply an embedding layer to this flattened representation, which is represented by the following formula:

(1) $$\begin{array}{c} H_{1}^{\prime }=flatten\left(H_{1}\right) \end{array}$$

(2) $$\begin{array}{c} e_{H_{1}^{\prime }}=embedding\left(H_{1}^{\prime }\right) \end{array}$$

The flatten function is often used in deep learning to reshape $H_{1}$ into a one-dimensional vector of length L×M. Another useful tool is the embedding function, which maps discrete values to flat trainable vector representations. In this case, $e_{H_{1}^{\prime }}\in R^{L\times M,d}$ represents the embedded representation of $H_{1}$. *d* represents the dimension of the mapped vector after embedding.

We transform $e_{H_{1}^{\prime }}$ to $R^{L\times M\times d}$ by reversing its size. This allows us to investigate the temporal patterns of users’ historical behavior, both short-term and long-term. Next, we feed $e_{H_{1}^{\prime }}$ into the LSTM. Let us take a subsequence $e_{H_{1}^{\prime }}^{l}$ from $e_{H_{1}^{\prime }}$ as an example, where l<L. The LSTM calculation process is described by the following formulas.

(3) $$\begin{array}{c} i_{t}=\sigma \left(W_{i}\cdot \left[h_{t-1},e_{H_{1}^{\prime },t}^{l}\right]+b_{i}\right) \end{array}$$

(4) $$\begin{array}{c} f_{t}=\sigma \left(W_{f}\cdot \left[h_{t-1},e_{H_{1}^{\prime },t}^{l}\right]+b_{f}\right) \end{array}$$

(5) $$\begin{array}{c} \tilde{C_{t}}=tanh\left(W_{C}\cdot \left[h_{t-1},e_{H_{1}^{\prime },t}^{l}\right]+b_{C}\right) \end{array}$$

(6) $$\begin{array}{c} o_{t}=\sigma \left(W_{o}\cdot \left[h_{t-1},e_{H_{1}^{\prime },t}^{l}\right]+b_{o}\right) \end{array}$$

(7) $$\begin{array}{c} C_{t}=f_{t}*C_{t-1}+i_{t}*\tilde{C_{t}} \end{array}$$

(8) $$\begin{array}{c} h_{t}=o_{t}*tanh\left(C_{t}\right) \end{array}$$

**Figure 3:**
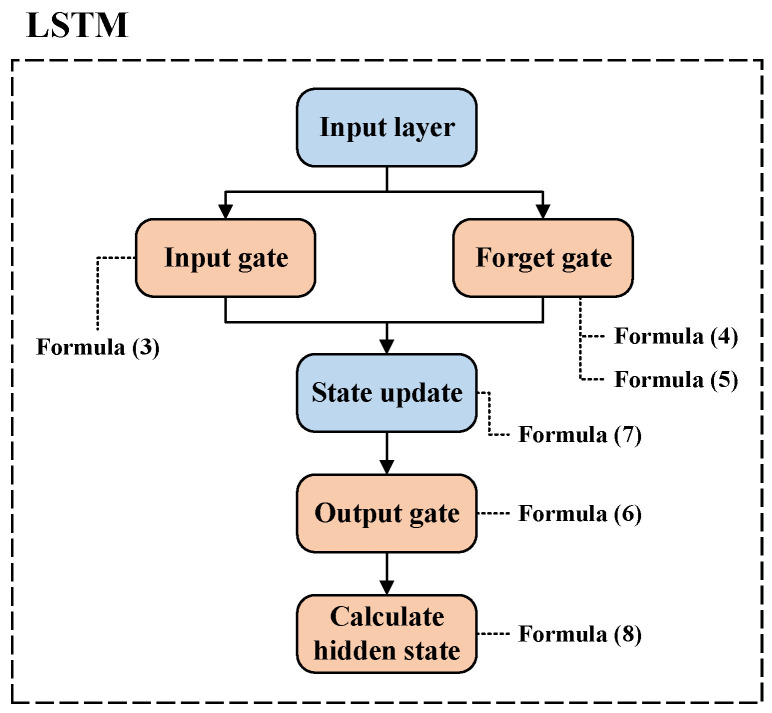
LSTM processing flow.

Formula (3) represents the input gate, Formulas (4) and (5) represent the forget gate, Formula (6) corresponds to the output gate, Formula (7) represents the long-term memory in the LSTM, and Formula (8) represents the short-term memory. The input layer of LSTM primarily receives a sequence of data. Each time step in the LSTM unit corresponds to an element in the sequence. Subsequently, LSTM inputs the results from the input layer to the input gate and forget gate. For the input gate, it calculates the output by constructing a weight matrix and applying an activation function, as shown in Formula (3). This gate is mainly responsible for controlling the amount of new information entering the new state. The forget gate operates similarly to the input gate, using trainable weight matrices and an activation function to compute outputs, as shown in Formulas (4) and (5). This gate is primarily responsible for controlling the information from the previous state that is forgotten. LSTM then utilizes Formula (7) to merge the results obtained from Formulas (3)–(5). Subsequently, the output gate, represented by Formula (6), controls how much information from the updated state will flow into the state at the next time step. Finally, the results obtained from the output gate are input into Formula (8) to calculate the hidden state, which represents the information flowing from each time step to the next. The specific process is illustrated in Figure 3.

The variable $e_{H_{1}^{\prime }}^{l}$ denotes the historical interaction behavior of the *l*-th sequence in $e_{H_{1}^{\prime }}$ at time *t*. Additionally, $h_{t-1}$ denotes the hidden state; $i_{t}$ denotes the output of the input gate; $f_{t}$ and $\tilde{C_{t}}$ represent the output of the forget gate; $o_{t}$ is the output of the output gate; $\left\{W_{i},W_{f},W_{C},W_{o}\right\}$ are the trainable parameters for each gate; $\left\{b_{i},b_{f},b_{C},b_{o}\right\}$ are the biases of each gate; σ represents the activation function.

**Figure 4:**
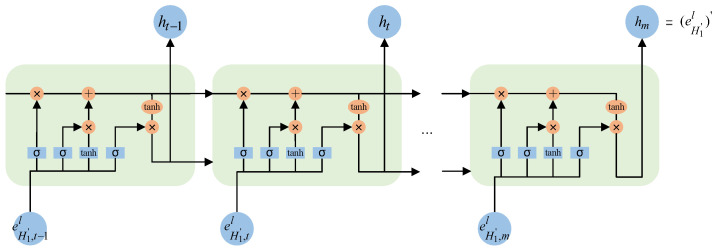
Extracting long and short-term interests of users.

The LSTM algorithm processes the results obtained from these gates through a specific formula to derive a hidden state denoted as $h_{t}$. This hidden state contains both long- and short-term memory information. At this point, $e_{H_{1}^{\prime },t}^{l}$ at time *t* has been transformed into $h_{t}$. These operations are performed at each time point, allowing the model to extract the temporal dynamics of a user’s historical behavior sequence, thereby uncovering their long and short-term interests. Finally, through LSTM, $e_{H_{1}^{\prime }}$ is transformed into $e_{H_{1}^{\prime }}^{\prime }$, where $e_{H_{1}^{\prime }}^{\prime }\in R^{L\times d}$. Refer to Figure 4 for a visual illustration of the specific process and Algorithm 1 for a detailed algorithmic flow.
**Algorithm 1** Long short-term interest representation of users’ historical behavior sequences**Require:** 
the users’ historical behavior sequence $H_{1}$**Ensure:** 
History behavior embedding $e_{H_{1}^{\prime }}^{\prime }$ with user long and short-term interests. 1:Obtain the flattened users’ historical behavior sequence $H_{1}^{\prime }$ through Formula (1) 2:Obtain the embedding matrix $e_{H_{1}^{\prime }}$ through Formula (2) 3:Initialize the parameters $\left\{W_{i},W_{f},W_{C},W_{o},b_{i},b_{t},b_{C},b_{o}\right\}$ of the LSTM 4:**for** l=1toL **do** 5:    **for** t=1toM **do** 6:        Input $e_{H_{1}^{\prime },t}^{l}$ 7:        Obtain $h_{t}$ according to Formulas (3)–(8) 8:    **end for** 9:**end for**10:Obtain an embedding matrix $e_{H_{1}^{\prime }}^{\prime }$ with long and short-term interests of users11:return $e_{H_{1}^{\prime }}^{\prime }$

#### 3.5.2. Time-Dynamic Representation of Similarity Relationships among Remote Sensing Resource Categories

This module requires two inputs: the categories to which remote sensing resources belong ($H_{2}$) and the historical behavior sequence ($H_{1}$). It should be noted that each remote sensing resource may belong to multiple categories, and the co-occurrence frequency of categories may vary among all remote sensing resources, which means that the similarity between categories may differ. To address this, we construct a matrix $A^{times}$ with the co-occurrence frequency of any two categories as its elements. Next, we use CF to obtain the cosine similarity between categories according to this matrix. The resulting similarity matrix is denoted as $A^{s}$. The calculation process is shown in the following formula:

(9) $$\begin{array}{c} A_{v_{i}v_{j}}^{s}=cosine\_similarity\left({\left(A^{times}\right)}^{T}\right)=\frac{{\left(A^{times}\right)}_{i}^{T}\cdot {\left(A^{times}\right)}_{j}^{T}}{∥{\left(A^{times}\right)}_{i}^{T}∥\cdot ∥{\left(A^{times}\right)}_{j}^{T}∥} \end{array}$$

The notation $A_{v_{i}v_{j}}^{s}$ refers to the similarity between categories $v_{i}$ and $v_{j}$. The symbol ${\left(A^{times}\right)}^{T}$ represents the transpose of the matrix $A^{times}$. ${\left(A^{times}\right)}_{i}^{T}$ is the *i*-th column of the matrix and ${\left(A^{times}\right)}_{j}^{T}$ is the *j*-th column. Here, *i* and *j* can represent any pair of categories.

We obtain the similarity matrix $A^{s}$ of categories through the process mentioned above. To further utilize the significance of this similarity, we use an MLP in combination with this matrix for initializing the embedding of remote sensing resource categories. This embedding method controls the initial distribution of feature vectors, making sure that they are not randomized but rather carry initial similarity information. The calculation process for this is shown in the following formulas.

(10) $$\begin{array}{c} e_{A^{s}}^{\left(1\right)}=\sigma \left(W_{s}^{\left(1\right)}A^{s}+b_{s}^{\left(1\right)}\right) \end{array}$$

(11) $$\begin{array}{c} e_{A^{s}}^{\left(l\right)}=\sigma \left(W_{s}^{\left(l\right)}{\left(A^{s}\right)}^{\left(l-1\right)}+b_{s}^{\left(l\right)}\right) \end{array}$$

(12) $$\begin{array}{c} e_{A^{s}}=\sigma \left(W_{s}^{\left(L\right)}{\left(A^{s}\right)}^{\left(L-1\right)}+b_{s}^{\left(L\right)}\right) \end{array}$$

**Figure 5:**
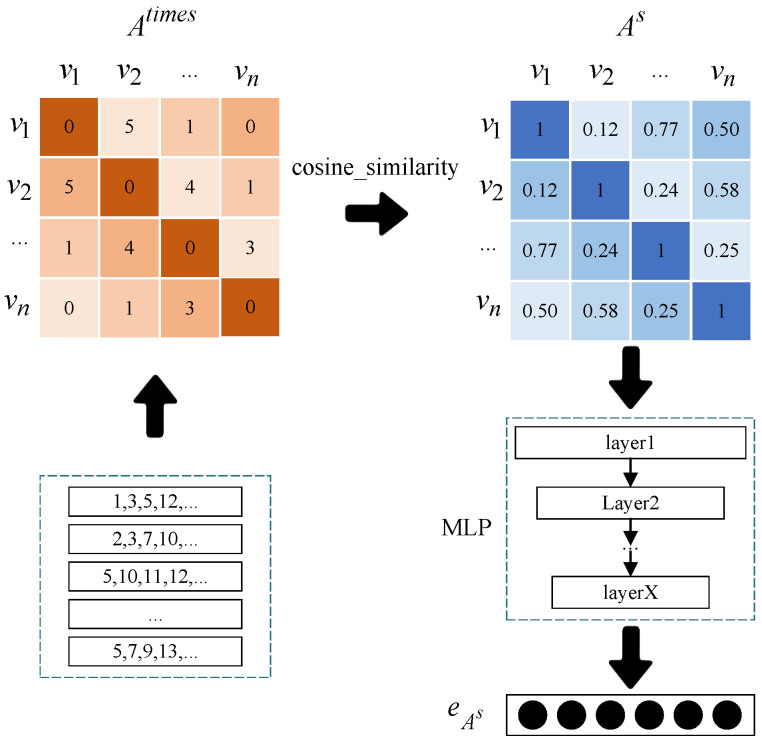
Similarity embedding matrix extraction.

Formula (10) represents the input layer of the MLP, Formula (11) describes the hidden layers, and Formula (12) describes the output layer. Here, *l* is the layer number, and *L* denotes the total number of layers, where 1<l<L. The trainable network weights in the input, hidden, and output layer are denoted by $\left\{W_{s}^{\left(1\right)},W_{s}^{\left(l\right)},W_{s}^{\left(L\right)}\right\}$, respectively. The network biases in each layer are represented by $\left\{b_{s}^{\left(1\right)},b_{s}^{\left(l\right)},b_{s}^{\left(L\right)}\right\}$. Additionally, the outputs in each layer are represented by $\left\{e_{A^{s}}^{\left(1\right)},e_{A^{s}}^{\left(l\right)},e_{A^{s}}\right\}$, where $e_{A^{s}}$ represents the category feature representation with similarity information after transformation. The activation function, represented by σ, is the same across all layers. The specific operation is illustrated in Figure 5.

We complete the initialization of embeddings for remote sensing resource categories by following the steps mentioned above. To construct a category interaction graph structure $G^{s}$, we utilize the co-occurrence relationship among categories. We take the categories to which remote sensing resources belong as nodes. If two categories of remote sensing resources co-occur, we consider that there is an adjacency relationship between these two categories, meaning there is an edge relationship. By analysing each type of remote sensing resource, we can obtain the edge relationships between all categories, thereby constructing the interaction graph $G^{s}$. We also use GCN to propagate messages and aggregate these embeddings and graph structures. This helps us obtain category embeddings $e_{A^{s}}^{\prime }$ with similarity relationships. The computational process is depicted in the following formulas.

(13) $$\begin{array}{c} e_{A^{s}}^{\left(l+1\right)}=\sigma \left({\left(\hat{D^{-\frac{1}{2}}}\hat{A}\hat{D^{-\frac{1}{2}}}\right)}_{G^{s}}e_{A^{s}}^{\left(l\right)}W_{G^{s}}^{\left(l\right)}\right) \end{array}$$

(14) $$\begin{array}{c} \hat{A_{G^{s}}}=A_{G^{s}}+I_{G^{s}} \end{array}$$

(15) $$\begin{array}{c} \hat{{\left(D_{G^{s}}\right)}_{ii}}=\sum_{j=0}^{\left|V\right|}\hat{{\left(A_{G^{s}}\right)}_{ij}} \end{array}$$

Here, *l* represents the layer of GCN where 0<l<L. $e_{A^{s}}^{\prime }$ is equivalent to $e_{A^{s}}^{\left(L\right)}$ when l+1=L. $\hat{A_{G^{s}}}$ represents the graph structure. $I_{G^{s}}$ is an identity matrix. $\hat{D_{G^{s}}}$ is a diagonal matrix representing the node degrees. $\hat{D^{-\frac{1}{2}}}$ normalizes $\hat{A_{G^{s}}}$. $\hat{{\left(D_{G^{s}}\right)}_{ii}}$ is the degree of the *i*-th node, obtained by summing the *i*-th row of $\hat{D_{G^{s}}}$ or the *i*-th column of $\hat{A_{G^{s}}}$. α represents the activation function, and to this end, we use the ReLU.

We obtain category embeddings $e_{A^{s}}^{\prime }$ with similarity relationships through the above operations. In order to capture the temporal dynamics of these similarities, we use the historical behavior sequence $H_{1}$ to form a sequence $H_{1}^{s}$ with similarity relationships. Then, we use LSTM to encode the temporal characteristics of the sequence $H_{1}^{s}$, which is demonstrated in the following formulas.

(16) $$\begin{array}{c} e_{H_{1}^{s}}=LSTM\left(H_{1}^{s}\right) \end{array}$$

In Section 3.5.1, we introduce the detailed calculation process of LSTM. Therefore, in this section, we use LSTM to represent it. The embedded representation with temporal dynamics of similarity relationships captured by LSTM is denoted by $e_{H_{1}^{s}}$. It is important to note that since these two LSTMs are distinct, their network parameters are also different. The algorithmic process for this part is shown in Algorithm 2.

#### 3.5.3. Representation of Potential Influencing Factors for Unique User Identification ID

This module takes $H_{3}$ as input. We embed user ID sequences as the initialized representation, which is denoted as $e_{H_{3}}\in R^{L\times d}$. The calculation formula is shown as follows:

(17) $$\begin{array}{c} e_{H_{3}}=embedding\left(H_{3}\right) \end{array}$$

These three modules provide us with embeddings that capture the user’s temporal interests $e_{H_{1}^{\prime }}^{\prime }\in R^{L\times d}$, time-dynamic similarity relationships $e_{H_{1}^{s}}\in R^{L\times d}$, and latent information about the user’s unique identification $e_{H_{3}}\in R^{L\times d}$.

### 3.6. Concatenation Layer

This layer connects the embeddings from the three modules to create the final combined embedding representation, $e_{f}$, where $e_{f}\in R^{L\times 3d}$. The connection operation is formulated as follows:

(18) $$\begin{array}{c} e_{f}=concat\left(e_{H_{1}^{\prime }}^{\prime },e_{H_{1}^{s}},e_{H_{3}}\right)=e_{H_{1}^{\prime }}^{\prime }\left|\right|e_{H_{1}^{s}}\left|\right|e_{H_{3}} \end{array}$$

where concat() denotes the concatenation function. This function concatenates $e_{H_{1}^{\prime }}^{\prime }$, $e_{H_{1}^{s}}$, and $e_{H_{3}}$ along the last dimension.
**Algorithm 2** Time-dynamic representation of similarity relationships among remote sensing resource categories**Require:** 
the users’ historical behavior sequence $H_{1}$; the remote sensing resource category $H_{2}$**Ensure:** 
Embedding $e_{H_{1}^{s}}$ with Time-Dynamic Similarity Graph Structure Relationships. 1:Generating the co-occurrence rate matrix $A^{times}$ based on $H_{2}$ 2:Obtain the embedding matrix $e_{H_{1}^{\prime }}$ through Formula (2) 3:Initialize the similarity matrix $A^{s}$ 4:**for** i=0to|V| **do** 5:    **for** j=0to|V| **do** 6:        **if** i=j **then** 7:           $A_{v_{i}v_{j}}^{s}=0$ 8:        **else** 9:           $A_{v_{i}v_{j}}^{s}=cosine\_similarity\left({\left(A^{times}\right)}^{T}\right)$10:        **end if**11:    **end for**12:**end for**13:Initialize network parameters $\left\{W_{s}^{\left(1\right)},W_{s}^{\left(l\right)},W_{s}^{\left(L\right)},b_{s}^{\left(1\right)},b_{s}^{\left(l\right)},b_{s}^{\left(L\right)}\right\}$14:Feed $A^{s}$ to the MLP15:Obtain the embedding $e_{A^{s}}$ for the similarity matrix according to Formulas (10)–(12)16:Construct the adjacency graph structure $G^{s}$ based on $H_{2}$17:Initialize network parameters $W_{G^{s}}^{\left(l\right)}$18:$e_{A^{s}}$ and $G^{s}$ into GCN19:Obtain $e_{A^{s}}$ according to Formulas (13)–(15)20:Obtain $H_{1}^{s}$ based on $H_{1}$ and $e_{A^{s}}$21:Obtain $e_{H_{1}^{s}}$ based on Formula (16)22:return $e_{H_{1}^{s}}$

### 3.7. Multilayer Perceptron Layer

The MLP layer is used to convert $e_{f}$ into scores for all categories of remote sensing resources. The computation process is shown in the following formulas.

(19) $$\begin{array}{c} {\left(e_{f}^{\prime }\right)}^{\left(1\right)}=\sigma \left(W_{f}^{\left(1\right)}e_{f}+b_{f}^{\left(1\right)}\right) \end{array}$$

(20) $$\begin{array}{c} {\left(e_{f}^{\prime }\right)}^{\left(l\right)}=\sigma \left(W_{f}^{\left(l\right)}e_{f}^{\left(l-1\right)}+b_{f}^{\left(l\right)}\right) \end{array}$$

(21) $$\begin{array}{c} e_{f}^{\prime }=\sigma \left(W_{f}^{\left(L\right)}e_{f}^{\left(L-1\right)}+b_{f}^{\left(L\right)}\right) \end{array}$$

The current layer is denoted as *l* and the previous layer is denoted as l−1. The last layer is denoted as *L* and the second-to-last is denoted as L−1. The output of these layers is denoted as $\left\{{\left(e_{f}^{\prime }\right)}^{\left(1\right)},{\left(e_{f}^{\prime }\right)}^{\left(l\right)},e_{f}^{\prime }\right\}$, where $e_{f}^{\prime }\in R^{L\times |V|}$ and *e* is the final output. The trainable network parameters in each layer are represented by $\left\{W_{f}^{\left(1\right)},W_{f}^{\left(l\right)},W_{f}^{\left(L\right)}\right\}$, with $W_{f}^{\left(1\right)}\in R^{3d\times h^{\left(1\right)}}$, $W_{f}^{\left(l\right)}\in R^{h^{\left(l-1\right)}\times h^{\left(l\right)}}$, and $W_{f}^{\left(L\right)}\in R^{h^{\left(l\right)}\times d}$. The network biases in each layer are denoted by $\left\{b_{f}^{\left(1\right)},b_{f}^{\left(l\right)},b_{f}^{\left(L\right)}\right\}$, with $b_{f}^{\left(1\right)}\in R^{h^{\left(1\right)}\times 1}$, $b_{f}^{\left(l\right)}\in R^{h^{\left(l\right)}\times 1}$, and $b_{f}^{\left(L\right)}\in R^{d\times 1}$.

### 3.8. Output Layer

The output layer is a crucial component that converts the final result, denoted by $e_{f}^{\prime }$, obtained from the MLP layer into the probability of the embedding belonging to each remote sensing resource category using the Softmax layer. This layer, along with the loss in the optimization function, facilitates the model update. For instance, the calculation process of the *i*-th element in ${\left(e_{f}^{\prime }\right)}_{i}$ is demonstrated in the following formulas.

(22) $$\begin{array}{c} {\left(e_{f}^{\prime \prime }\right)}_{i}=Softmax\left({\left(e_{f}^{\prime }\right)}_{i}\right)=\frac{E^{{\left(e_{f}^{\prime }\right)}_{i}}}{\sum_{j=0}^{\left|V\right|}{\left(e_{f}^{\prime }\right)}_{ij}} \end{array}$$

After applying the Softmax transformation, ${\left(e_{f}^{\prime \prime }\right)}_{i}\in R^{1\times |V|}$ represents a probability matrix, while $e_{f}^{\prime \prime }\in R^{L\times |V|}$ represents the probability matrix corresponding to $e_{f}^{\prime }$. Here, ${\left(e_{f}^{\prime }\right)}_{ij}\in R^{1}$ is the *j*-th element in the *i*-th row.

### 3.9. Optimization

After obtaining the probability matrix $e_{f}^{\prime \prime }$, we use the cross-entropy loss to optimize our model. The formula for this loss is as follows.

(23) $$\begin{array}{c} loss=CrossEntropyLoss\left(e_{f}^{\prime \prime },t_{f}\right)=-\frac{1}{L}\sum_{j=0}^{L}\sum_{i=0}^{\left|V\right|}{\left(t_{f}\right)}_{ji}log{\left(e_{f}^{\prime \prime }\right)}_{ji} \end{array}$$

where $t_{f}$ represents the result in the real dataset, $t_{f}\in R^{L\times 1}$.

### 3.10. Deployment of the Recommendation Algorithm

**Figure 6:**
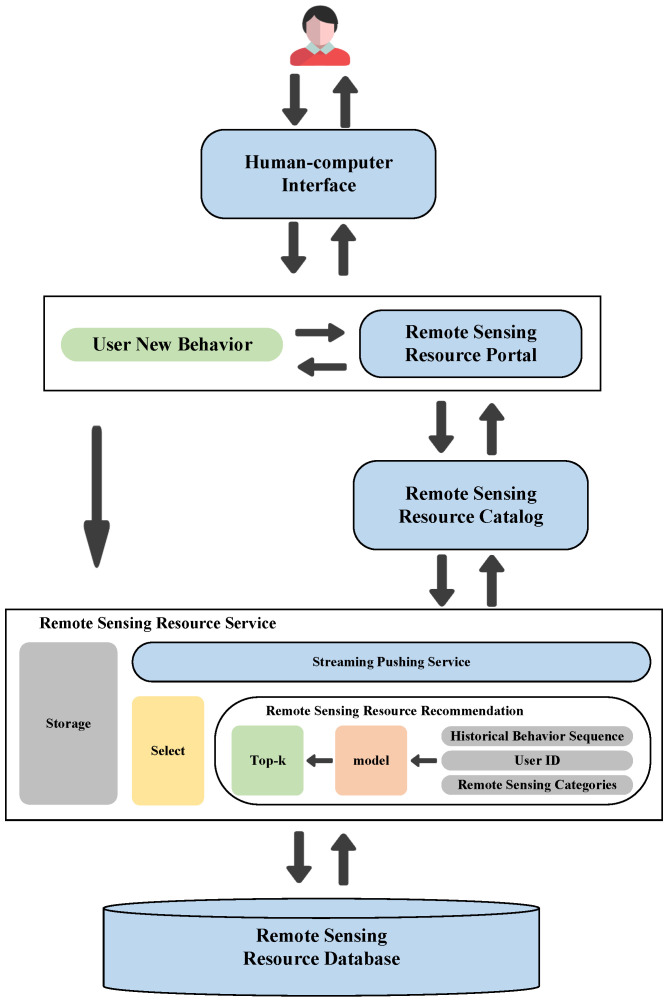
Recommendation algorithm deployment process.

In the previous sections, we have implemented personalized recommendations for remote sensing resources. By analyzing users’ temporal interests, identifying the similarity between remote sensing resource categories, and considering users’ unique potential factors, we have not only improved the recommendation performance but also moved away from the traditional filtering-based push of remote sensing resource service systems. We have designed a remote sensing resource streaming push service based on the TGDL-RSSR algorithm. This service includes three main modules. Figure 6 describes the specific process.

The first module obtains the ID of terminal system users, their search history, remote sensing resource categories, and relationships between categories. It then processes and analyzes the data. The second module deploys our TGDL-RSSR recommendation algorithm. The third module of our system involves a streaming push service that follows the traditional B/S architecture. The system includes two parts—the application side and the service side. On the application side, the current user’s ID is obtained and the content is displayed based on the service end’s response. The user’s new behavior is then stored in the database. On the service side, users’ recent history records and the classification information of remote sensing resources are retrieved from the database. The data are then analyzed and processed based on the model’s data format requirements. The TGDL-RSSR recommendation algorithm is deployed on the service end to generate recommendations based on the retrieved and processed data and user ID information. Finally, the results are sent to the application end through the streaming push service.

**Figure 7:**
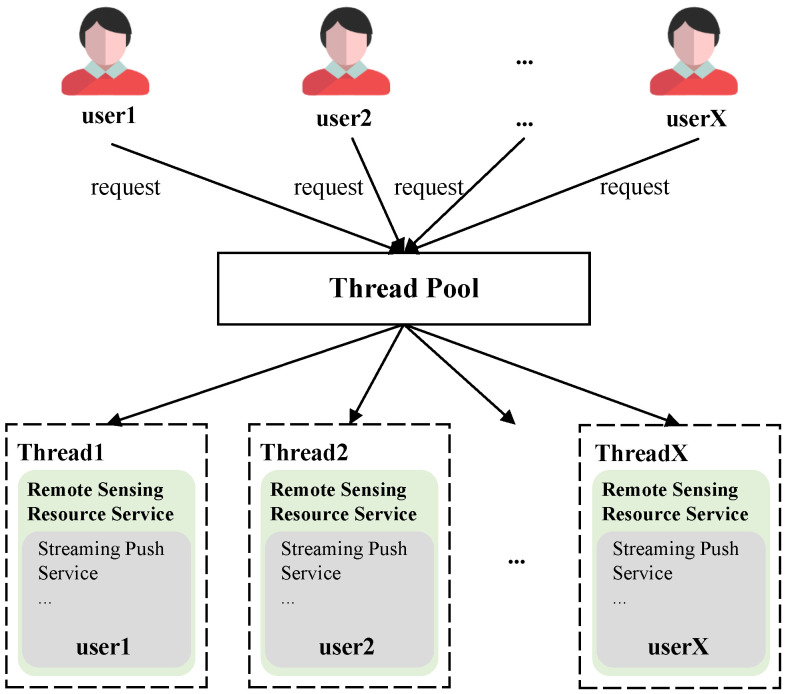
Multi-threading support in remote sensing resource service.

As the number of users increases, single-threaded tasks may struggle to keep up with the demands of multiple users. This can result in blocking issues, causing longer wait times and decreasing the efficiency of recommendations, ultimately impacting the user experience. To overcome this problem, we have implemented multi-threading support on the server side. When multiple users send requests simultaneously, threads can intelligently allocate these requests, with each thread independently handling a subset of requests. This significantly reduces the occurrence of blocking issues and ensures a smoother experience for all users. The visual representation of the multi-threaded scenario is shown in Figure 7.

## 4. Experiments

In this section, we verify the effectiveness of TSDL-RSSR by comparing it with other methods. The comparison is divided into two parts: (1) experiments involving three recommender system datasets; and (2) experiments involving a remote sensing service dataset. We compare our approach with several existing methods such as CF [45] and NeuralCF [46], LSTM [47], AGCN [48], and DCF [49].

### 4.1. Experimental Setting

#### 4.1.1. Experimental Environment

We use the PyTorch framework [50] to implement our proposed method. The running environment is configured with a 12th generation Intel(R) Core(TM) i7-12700H 2300 MHz CPU and 32 GB of RAM.

#### 4.1.2. Public Recommendation Datasets

We use two publicly available recommender system datasets, MovieLens [51], Amazon-clothes [52] and Amazon-books [53], to validate the effectiveness of TGDL-RSSR. Furthermore, we validate the applicability of TGDL-RSSR by utilizing a dataset of remote sensing services that we created. Table 4 provides detailed descriptions of both datasets.

The MovieLens dataset is a popular movie rating dataset that contains four fields: user ID, movie ID, rating, and timestamp. It includes 6040 users, 3706 movies, and 1,000,208 interactions. Our approach treats the movies in the dataset as a type of remote sensing resource, with the dataset representing the interactions between users and these resources. The interactions are represented as ratings that reflect users’ evaluations of the remote sensing resources. To implement our method, we needed to process the dataset in specific ways. For the adjacency graph structure, we considered two remote sensing resources to have an adjacency relationship if the same user accessed them. Regarding the similarity matrix, we collected all remote sensing resources visited by each user, treating this situation as a co-occurrence, and aggregated all users’ situations.

The Amazon-clothes dataset is a collection of clothing ratings that includes four fields: user ID, clothing ID, rating, and timestamp. We have treated clothing as a type of remote sensing resource category and processed this dataset in a similar way to the MovieLens dataset. The Amazon-clothes dataset has 4993 users, 39 different types of clothing, and a total of 6201 interactions.

The Amazon Books dataset is a book rating dataset with multiple fields, including user ID, book ID, review ID, review text, review timestamp, and more. For our method, we will exclude fields other than user ID, book ID, rating, and review timestamp. Additionally, we will convert the review timestamp to a timestamp format. Similar to the previous two datasets, we treat books as a category of remote sensing resources, and the specific processing method is the same as before. This dataset comprises 47,400 users, 36,412 books, and 154,555 interactions.

#### 4.1.3. Remote Sensing Service Dataset

**Figure 8:**
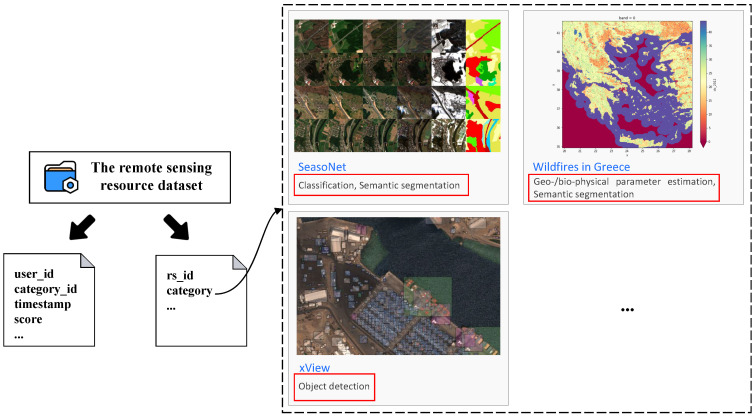
The remote sensing service dataset.

We have created a remote sensing service dataset by collecting the historical search records, user ID information, and type of remote sensing resources used by our users. The dataset is divided into two subsets—one that stores users’ historical behaviors and the other that stores the category information of remote sensing resources. When users search for remote sensing resources, the categories to which each resource belongs are displayed. Each resource may belong to multiple categories, and any combination of these categories may co-occur multiple times, resulting in higher similarity between them. The dataset comprises 300 users and 50 categories of remote sensing resources. Figure 8 provides a detailed illustration of the specific composition of this dataset.

In Figure 8, there are two sub-datasets. The first sub-dataset has four fields: user_id, category_id, timestamp, and score. These fields represent the user ID, the index representation of the category to which the resource belongs, the rating for the remote sensing resource category, and the timestamp of the user’s historical behavior, respectively. The second dataset also has two fields: rs_id and category. These fields represent the ID of the remote-sensing resource and all categories to which the remote-sensing resource belongs.

#### 4.1.4. Data Preprocessing

To partition the relationship dataset, we employ leave-one-out to split the dataset into training and testing sets. The training set is used for model training, while the testing set is used to predict and recommend user preferences after training the model, as well as to calculate the corresponding recommendation performance. To enhance the model’s ability to analyze user preferences during the dataset processing, we remove users with interaction history sequences involving remote sensing resources less than 10 times. This ensures that users used for model training have rich interaction histories.

#### 4.1.5. Parameter Settings

To standardize the embedding dimensions for the user and remote sensing service categories, we designated them as 50, aligning with the dimensions employed in comparable baseline methods. The MLP hidden layer incorporates three layers, consistent with the setup of other baseline methods. In the model architecture, the GCN consists of two layers, with an embedding dimension set at 50. The GCN layers for the AGCN method are configured identically. The learning rate for the two public datasets is set at 0.001, while for the remote sensing resource dataset, it is established at 0.005. As for other baseline methods, their learning rates fall within the range of 0.001, 0.005, and 0.01.

For model training, we utilize the Adam optimizer [54] for optimization. The iteration count is set to 100 for the MovieLens dataset, 70 for the Amazon-clothes dataset, 30 for the Amazon-books dataset, and 50 for the remote sensing resource dataset.

#### 4.1.6. Evaluation Metrics

We use three metrics to evaluate different methods, including recall@k, precision@k, and F1-score@k, represented by the following formulas.

(24) $$\begin{array}{c} Recall@k=\frac{1}{N}\sum_{u}^{N}\frac{\left|S_{u}\left(k\right)\cap T_{u}\right|}{\left|T_{u}\right|} \end{array}$$

(25) $$\begin{array}{c} Precision@k=\frac{1}{N}\sum_{u}^{N}\frac{\left|S_{u}\left(k\right)\cap T_{u}\right|}{k} \end{array}$$

(26) $$\begin{array}{c} F1@k=\frac{2*Recall@k*Precision@k}{Recall@k+Precision@k} \end{array}$$

$S_{u}\left(k\right)$ denotes the top *k* categories with the highest scores in the candidate set, and $T_{u}$ represents the resource categories in the test set. *N* denotes the total number of users.

### 4.2. Experiments on Recommender System Datasets

The section focuses on comparing the performance of the TGDL-RSSR model with other baseline models on recommended system datasets by adjusting hyperparameters and performing ablation experiments.

#### 4.2.1. Overall Comparison with Baseline Methods

Table 5 shows the results of the recommendation performance compared with the baseline models, from which we can draw the following conclusions.

Table 5 shows that our method outperforms other models on both the MovieLens, Amazon-clothes and Amazon-books datasets.

Traditional collaborative filtering models rely on shallow linear relationships between features and targets, which means they often overlook other important information. Consequently, their recommendation performance on the MovieLens, Amazon-clothes and Amazon-books datasets is not satisfactory. From the perspective of remote sensing resources, there is a high probability of recommending incorrect categories to users. On the other hand, the NCF model, which builds on CF, uses deep learning for optimization. By taking advantage of the powerful nonlinear capabilities of deep learning, NCF explores complex relationships between features and targets, resulting in a better recommendation performance than CF models. However, despite incorporating deep learning, NCF still lacks the ability to include other crucial information.

DCF uses deep learning to make improvements based on CF. It is similar to NCF in terms of incorporating the nonlinear computational capability of deep learning. However, DCF differs from NCF in its feature initialization method. In NCF, an embedding layer is used to initialize the IDs of users and items, whereas DCF uses MLP for user and item embedding initialization based on the rating matrix. The different initialization methods between NCF and DCF can lead to variations in the fit to the target rating matrix and consequently differences in recommendation performance.

LSTM has a unique long short-term memory function that helps it to mine the temporal interests of users effectively. It can identify hidden dynamic temporal factors within input time series information. By analyzing historical behavior sequences, the model is able to uncover users’ latent interests and provide recommendations for their next interest. Since LSTM is a form of deep learning, it possesses powerful nonlinear computational capabilities. As a result, it outperforms CF and NCF in terms of recommendation performance.

AGCN is an extension of GCN that incorporates an attention layer. It uses graph structure information to extract information for each node in the graph. This allows for efficient node propagation, making it easy to perceive adjacency relationships between nodes. Unlike GCN, which propagates only to neighbors equally, AGCN considers the impact level of each neighbor on that node, making it more practically significant. AGCN is a powerful nonlinear deep learning technique, similar to LSTM, and has demonstrated excellent performance in recommendation systems.

Our approach, TGDL-RSSR, not only considers the similarity of users’ behavior patterns but also considers the temporal dynamics of these patterns. We achieve this by incorporating the mining of similarity adjacency relationships and the temporal dynamics of relationships between item categories, using a graph structure and LSTM. Additionally, we model each user’s unique potential influencing factors using their unique identification ID to capture the users’ individual characteristics. As a result, our approach outperforms other models on the MovieLens, Amazon-clothes and Amazon-books datasets. Comparing our findings with the best results of baseline models, we can find that on the MovieLens, recall@5, precision@5, and F1-score@5 are higher by 2.76%, 2.96%, and 2.76%, respectively. Recall@10, precision@10, and F1-score@10 are higher by 4.63%, 4.47%, and 4.7%, respectively. Recall@15, precision@15, and F1-score@15 are higher by 4.08%, 3.94%, and 4.21%, respectively. Recall@20, precision@20, and F1-score@20 are higher by 13.82%, 13.68%, and 13.81%, respectively. On the Amazon-clothes, recall@5, precision@5, and F1-score@5 are higher by 10.65%, 10.41%, and 10.58%, respectively. Recall@10, precision@10, and F1-score@10 are higher by 10.08%, 10.31%, and 10.2%, respectively. Recall@15, precision@15, and F1-score@15 are higher by 6.44%, 6.56%, and 6.71%, respectively. Recall@20, precision@20, and F1-score@20 are higher by 5.88%, 6.36%, and 6.06%, respectively. Similarly, on the Amazon-books, recall@5, precision@5, and F1-score@5 are higher by 5.68%, 5.69%, and 5.76%, respectively. Recall@10, precision@10, and F1-score@10 are higher by 12.10%, 12.26%, and 12.00%, respectively. Recall@15, precision@15, and F1-score@15 are higher by 6.93%, 6.94%, and 6.96%, respectively. Recall@20, precision@20, and F1-score@20 are higher by 5.16%, 4.29%, and 4.51%, respectively.

**Figure 9:**
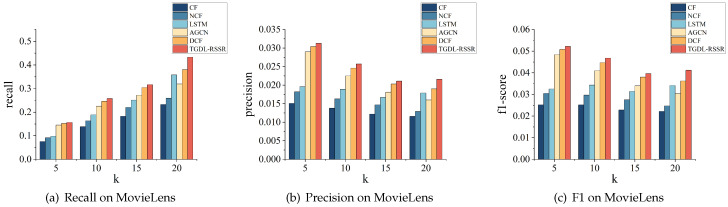
Performance comparison on MovieLens.

**Figure 10:**
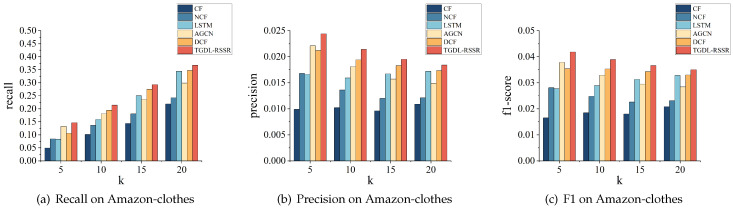
Performance comparison on Amazon-clothes.

**Figure 11:**
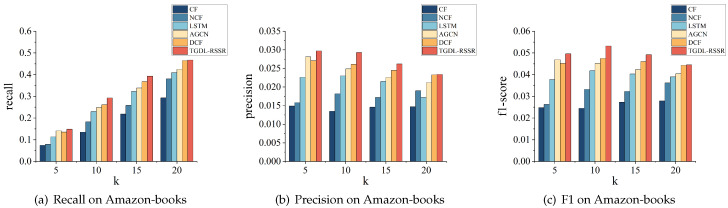
Performance comparison on Amazon-books.

A more intuitive representation is shown in Figure 9, Figure 10 and Figure 11.

#### 4.2.2. Sensitivity Analysis

This section explores the impact of different learning rates on recommendation performance, as well as the impact of different embedding sizes.

**Figure 12:**
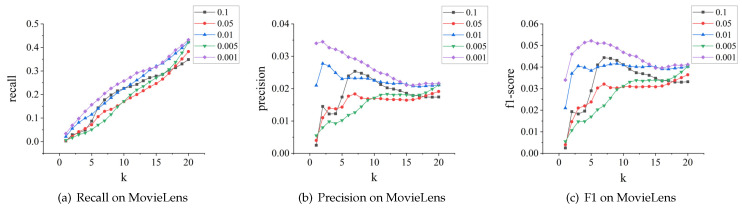
Parameter tuning for learning rate on MovieLens.

**Figure 13:**
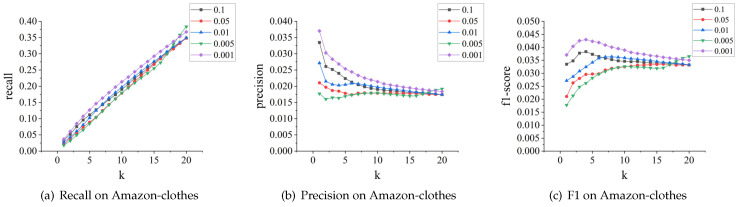
Parameter tuning for learning rate on Amazon-clothes.

**The effect of learning rate.** We tested different values of the learning rate, including 0.001, 0.005, 0.01, 0.05, and 0.1. The impact results on the MovieLens and Amazon-clothes datasets are shown in Figure 12 and Figure 13, respectively. We can see that the model’s recall, precision, and F1-score perform optimally when the learning rate is 0.001. Moreover, compared to other learning rates, the model’s recommendation performance remains relatively stable when the learning rate is at 0.001. However, when set at 0.1, the model’s performance on both datasets fluctuates significantly.

**Figure 14:**
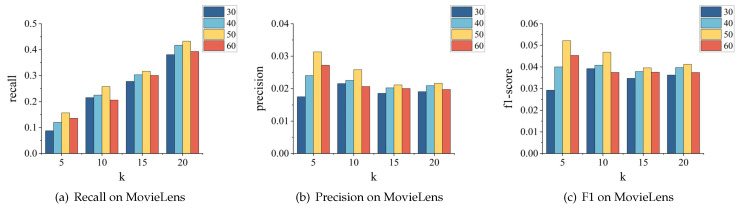
Parameter tuning for embedding dimension on MovieLens.

**Figure 15:**
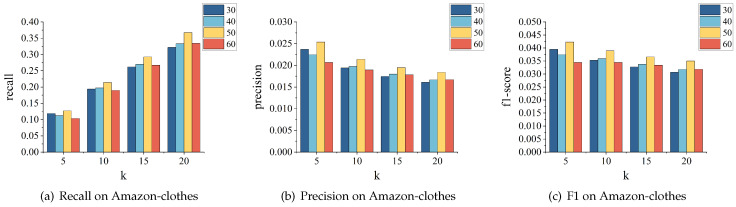
Parameter tuning for embedding dimension on Amazon-clothes.

**The effect of embedding dimension.** This section aims to demonstrate how embedding dimensions can affect the recommendation performance. We experimented with embedding dimensions of 30, 40, 50, and 60 and evaluated the model’s performance on the MovieLens and Amazon-clothes datasets, as shown in Figure 14 and Figure 15, respectively. The results indicate that the model performs best when the dimension is 50, as it achieves optimal recall, precision, and f1-score values. This suggests that bigger embedding dimensions might not lead to better performance as long as the appropriate value is selected to capture the necessary information.

#### 4.2.3. Ablation Study

**Figure 16:**
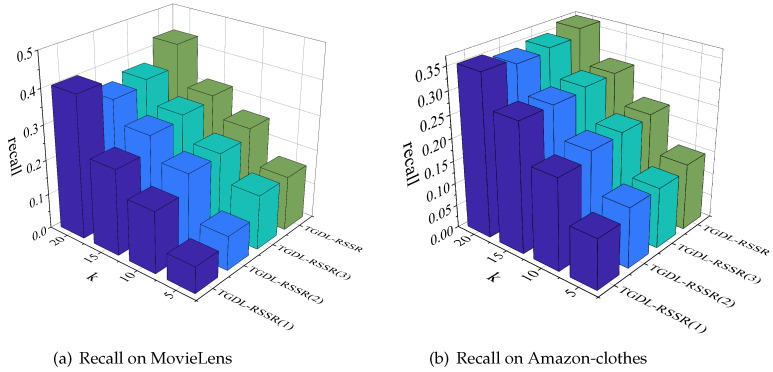
Ablation study on MovieLens and Amazon-clothes.

We conduct ablation experiments by selectively removing three key modules from the core layer. Figure 16a demonstrates the impact of each module on recall@k on the MovieLens, while Figure 16b illustrates the impact on the Amazon-clothes. In Figure 16, TGDL-RSSR(1), TGDL-RSSR(2), TGDL-RSSR(3), and TGDL-RSSR represent models with the first module (users’ historical sequence), the second module (resource category similarity graph structure), the third module (user unique identification ID), and the original model being removed, respectively.

For the metrics of recall@5, recall@10, recall@15, and recall@20, the results presented in Figure 16a demonstrate that TGDL-RSSR outperforms TGDL-RSSR(1) by 107.3%, 45.89%, 29.18%, and 6.13% respectively. TGDL-RSSR also surpasses TGDL-RSSR(2) by 59.69%, 13.94%, 8.95%, and 22.70% respectively. Furthermore, TGDL-RSSR exceeds TGDL-RSSR(3) by 0.32%, 10.75%, 4.46%, and 17.21% respectively. In Figure 16b, TGDL-RSSR surpasses TGDL-RSSR(1) by 32.26%, 14.14%, 3.28%, and 3.75% respectively. TGDL-RSSR also outperforms TGDL-RSSR(2) by 10.96%, 4.82%, 3.01%, and 7.09% respectively. Finally, TGDL-RSSR exceeds TGDL-RSSR(3) by 10.19%, 2.83%, 0.35%, and 4.75% respectively. These results indicate the importance of leveraging LSTM to explore the temporal dynamics to improve the recommendation performance. Furthermore, the CF+GCN+LSTM pattern is effective in exploring the temporal dynamics of similarity adjacency relationships between resources in the second module. Finally, modeling user-unique identification IDs using an embedding layer is effective in uncovering unique user characteristics in the third module.

In comparing the performance of TGDL-RSSR with TGDL-RSSR(1), we find that in scenarios where users have a more extensive history, TGDL-RSSR can more accurately analyze users’ long and short-term interests, thereby achieving more precise recommendations. Similarly, in comparison with TGDL-RSSR(2), if users’ search histories cover a diverse range of remote sensing resource categories, i.e., a higher co-occurrence frequency among resource categories, TGDL-RSSR’s analysis of the relationships and similarities between remote sensing resource categories will be clearer, leading to more accurate recommendations.

#### 4.2.4. Computational Efficiency

**Figure 17:**
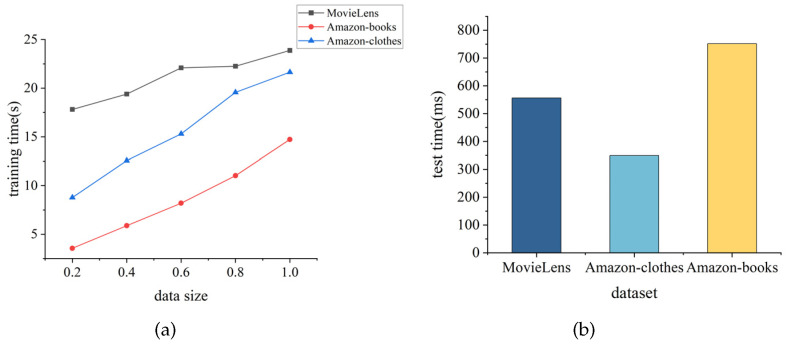
Computational efficiency on MovieLens, Amazon-clothes and Amazon-books. (**a**) Training time on MovieLens, Amazon-clothes and Amazon-books. (**b**) Test time on MovieLens, Amazon-clothes and Amazon-books.

Within this section, we explored the training and prediction time of TGDL-RSSR on datasets of different scales. For training time, we divided the dataset into proportions of 20%, 40%, 60%, 80%, and 100% to explore the training time of the model at different ratios. Each dataset followed the same procedure. For prediction time, we used the test set to obtain the model’s prediction time. The details are shown in Figure 17.

As shown in Figure 17a, when the computational resources (dataset) size changes, the model’s training time also increases. When the data scale is large, the computational time is significantly affected due to the higher complexity of relationships in the dataset, and vice versa. Additionally, even when maintaining the computational resources at the same scale, each dataset’s complexity differs, resulting in different training times at the same proportion. Although the model takes a relatively long time to reach the convergence point at a certain scale, the prediction time is relatively short (100% data proportion). In the MovieLens dataset, the model’s prediction time is 556.312 ms; in the Amazon-clothes dataset, it is 348.76 ms; in the Amazon-books dataset, it is 751.475 ms, meaning the model’s prediction time is less than 1 s for each dataset. This indicates that TGDL-RSSR performs well in terms of prediction time, even on large-scale datasets. Therefore, when deployed in a remote sensing resource information service system, the system can provide acceptable response times to users.

### 4.3. Experiments on Remote Sensing Service Dataset

This section conducts experiments on a real remote sensing resource dataset we created, to validate the effectiveness of our approach in comparison to traditional methods used in remote sensing service systems.

#### 4.3.1. Overall Comparison with Baseline Methods

**Figure 18:**
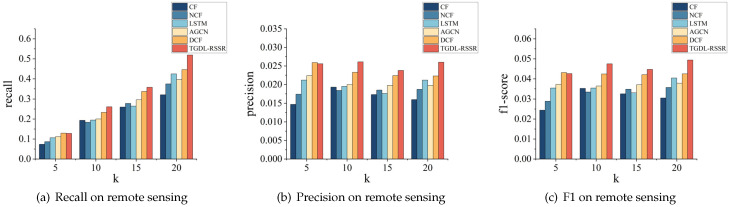
Performance comparison on remote sensing service dataset.

We compared the recommendation performance of TGDL-RSSR with other methods using the collected actual remote sensing resource dataset. The comparative results are described in Table 6. A more intuitive representation is shown in Figure 18.

In Section 4.2.1, we have analyzed the structural differences between different models. Here, we are going to compare the performance of TGDL-RSSR with the best-performing baseline model. Although TGDL-RSSR did not achieve the highest values for recall@5, precision@5, and F1-score@5 on the remote sensing resource dataset, it still outperformed the baseline model in recall@10, precision@10, and F1-score@10 by 12.07%, 12.02%, and 12.03% respectively. Moreover, it surpassed the baseline model in recall@15, precision@15, and f1-score@15 by 6.27%, 6.25%, and 6.18%, respectively. In recall@20, precision@20, and f1-score@20, our method exceeded the baseline model by 16.34%, 16.59%, and 16.24%, respectively. This comparison demonstrates the effectiveness of our approach.

#### 4.3.2. Usability Experiments

In this section, we explore the practicality of TGDL-RSSR by discussing the deployment process of the model, users’ historical behavior collection, and remote sensing resource category recommendations.

**Figure 19:**
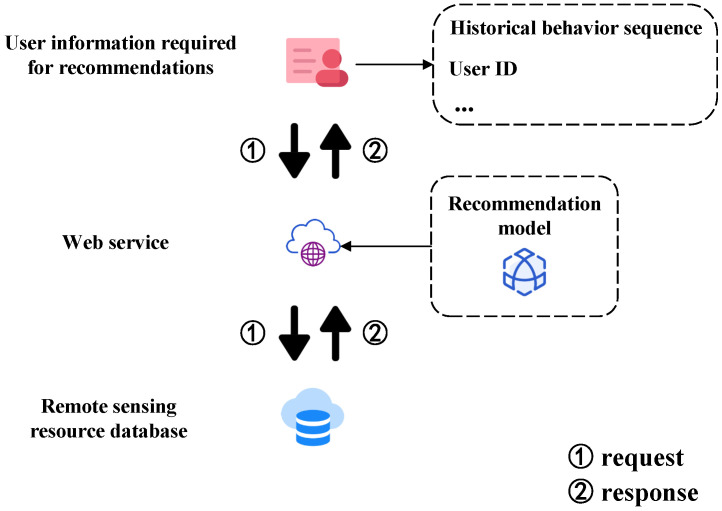
Deployment process of the model.

**Deployment process of the model.** First, let us discuss integrating the model into the system. In Figure 6, we describe deploying the remote sensing resource recommendation model as a web service on the server and returning its recommendations to users via stream push. Traditional remote sensing resource information service systems rely on users searching through keywords, with the server directly querying the database to display search results. For a recommendation model, it adds a recommendation model service between user-related information and the database, as illustrated in Figure 19.

**Figure 20:**
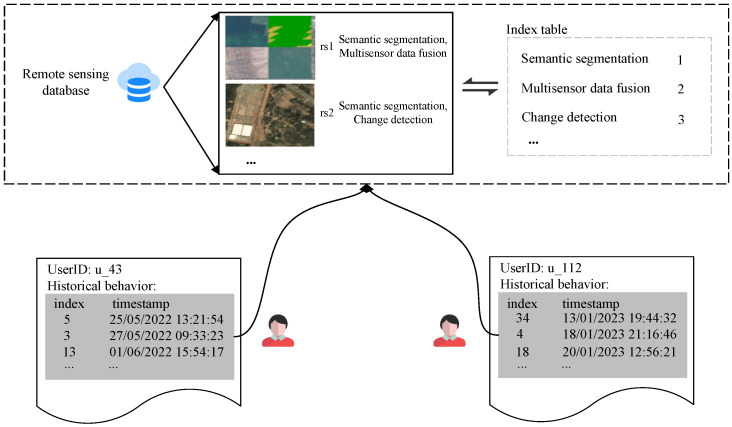
User historical behavior collection.

**Users’ historical behavior collection.** Then, we demonstrate the collection of historical behaviors for some users. We take the examples of two users, u_43 and u_112 (43 and 112 are their unique identification IDs). For u_43, we have collected a three-month history of remote sensing resource searches, while for u_112, we have collected a seven-month history of such searches. Since our recommendation method requires a user’s historical behavior, we have collected the behavior of these two users. During the collection process, we used the user’s most recent search behavior as the reference and collected nine historical behaviors in reverse chronological order, summing up to a total of eight historical behaviors. The collected results are shown in Figure 20.

We present relevant image samples, ID numbers, and their corresponding categories for each dataset of remote sensing resources in Figure 20. We use an index table to remap and index each remote sensing resource category. In the user box, we have illustrated the data structure used for recommendation, which includes the user ID and their historical behavior sequence. The historical behavior sequence for User 43 is {5, 3, 13, 5, 5, 5, 31, 13}, and for User 112, it is {14, 4, 18, 18, 14, 14, 4, 18}, where the values represent the indexed mapping of remote sensing resource categories.

**Figure 21:**
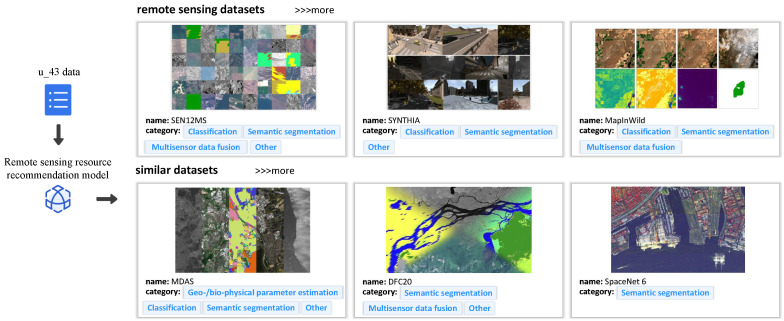
Recommendation results for u_43.

**Figure 22:**
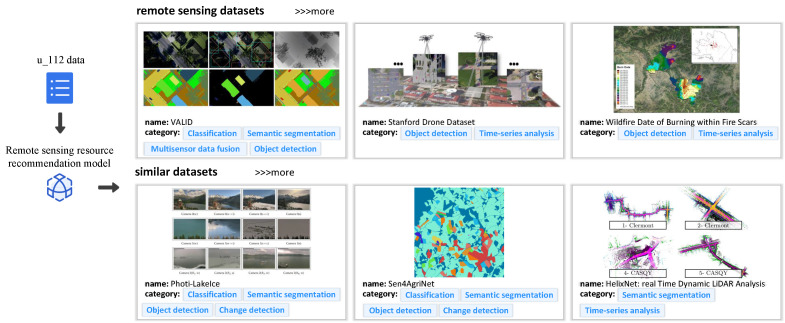
Recommendation results for u_112.

**Recommendation of remote sensing resource categories.** We showcase the recommendation results of our method based on the past behavior of two users, u_43 and u_112. Instead of recommending a single category, our method recommends a sequence of categories. The significance of similarity analysis in our method is validated by the categories in the sequence. You can find the recommendation results in Figure 21 and Figure 22. The remote sensing resources depicted in Figure 21 and Figure 22 are sourced from the literature [55,56,57,58,59,60,61,62,63,64,65,66].

For the recommendation method, we use binary stream push to return the model’s recommended results. For remote sensing resources, they are mainly composed of images. On the server side, if traditional image transmission methods are used, it may cause network congestion during the push, thereby increasing the duration of the push and affecting both the performance of the service system and the user experience. Stream push technology essentially involves processing the results that the server needs to return into binary streams using server-side languages. This approach can significantly reduce the server-side load and the size of transmitted data, thereby avoiding network congestion caused by large data volumes.

For example, let us consider u_43. Based on the historical behavior sequence, it is likely that the next area of interest for this user is remote sensing resource category 5, which is named “Classification” in the index. Figure 21 shows that our remote sensing resource recommendation method successfully suggested relevant resources in this category. The recommended resources show that the “Classification” category is often associated with other categories like “Semantic segmentation” and “Multisensor data fusion”, indicating a high degree of similarity between these categories. The “similar datasets” section in Figure 21 demonstrates that our recommendation method effectively suggests remote sensing resources based on these similar categories. This method enables users to explore other similar categories of remote sensing resources, thereby enhancing their understanding of remote sensing resources.

#### 4.3.3. User Satisfaction Comparison

We compare TGDL-RSSR with traditional content-based retrieval methods [67] used in remote sensing resource service systems. We specifically look at the approach presented in [1]. We measure user satisfaction levels across three categories: zero, one, and two. A score of zero represents dissatisfaction, one indicates moderate satisfaction, and two signifies complete satisfaction. Then, we also collect statistics on the average usage time of users in the system. A shorter average usage time suggests that users can quickly find the resources they want when using the system. Conversely, a higher average usage time indicates that the system may not conduct an in-depth analysis of user preferences, leading users to spend more time articulating their needs and searching for resources. Finally, we conducted statistics on the system latency time for both methods. A shorter latency indicates that the method has a quicker response time, enhancing the user experience. The comparison results are presented in Table 7.

According to Table 7, our remote sensing resource recommendation method has proven to be more effective in terms of user satisfaction than traditional content-based retrieval methods. At the same time, this also indicates that by analyzing the historical behavior sequences generated by users when searching for remote sensing data, personalized analysis of users’ long and short-term interests can be conducted, thereby achieving more precise recommendations. This reduces the time users spend analyzing their needs and searching each time they use the system. By analyzing the similarity between resource categories when users search for resources, it is possible to explore resources similar to user preferences. While ensuring user usage needs are met, users can also become acquainted with similar resources. Moreover, after deploying the TGDL-RSSR method into the remote sensing resource service system, there has been a reduction in response time, allowing for more real-time effects on user requests. This ultimately enhances user satisfaction.

## 5. Conclusions and Future Work

Our study introduces TGDL-RSSR, a method for recommending remote sensing information services. It utilizes a dual-LSTM network with time awareness and similarity graph learning. The method consists of five layers, with the representation layer being the core layer, which is divided into three main modules. In the first module, we employ LSTM to analyze the long- and short-term interests as well as the temporal dynamics in users’ historical behavior sequences. The second module uses a CF+MLP+GCN+LSTM approach to explore the similarity graph structure adjacency relationships among remote sensing resource categories and the temporal dynamics of these relationships. In the third module, we model the unique identification ID of users using an embedding layer to uncover users’ unique potential characteristics. Finally, an MLP is employed to obtain the probability that the user’s next interest belongs to each category, thereby completing the remote sensing resources recommendations.

During the data preprocessing stage, we imposed limitations on the interaction frequency in users’ historical behavior, potentially leading to a significant decline in recommendation effectiveness when the user interaction history is sparse. The cold start phenomenon can result in an extremely sparse user interaction history. Therefore, cold start data pose a challenge, and represent an issue to be addressed in our future work. In our approach, we employ a dual LSTM. LSTM is prone to problems such as gradient vanishing and exploding during training, making it less stable when capturing temporal interests in users’ historical behavior sequences. Additionally, LSTM relies on sequential computation, which may impact the training efficiency of the model to some extent. To tackle these issues, we plan to replace LSTM with Transformer [68]. The Transformer model, besides addressing some of the drawbacks associated with LSTM, incorporates inherent positional encoding and multi-head attention. This enables it to better differentiate information from different positions in users’ historical behavior sequences and focus on various elements in the sequence, thereby achieving a more precise exploration of user preferences.

## Figures and Tables

**Table 1:** Remote sensing methods.

Methods	Sensor Type	Application Area
Optical sensor	Visible and infrared spectrum	Environmental monitoring, Agriculture, Urban planning
Radar sensor	Microwave radiation	Natural disaster monitoring, Resource management
Thermal infrared sensor	Infrared spectrum	Surface temperature monitoring, Vegetation health assessment

**Table 2:** Traditional active service approaches.

Active Service Methods	Description	Examples
Catalog Searching	Utilizes library catalogs, databases	Library catalogs, academic databases
Surveys and Interviews	Engages in communication with domain experts or practitioners	Professionals, industry practitioners
Archival Research	Retrieves historical records, documents	Archives, historical reports
On-site Investigations	Conducts site visits, observes and records	Field inspections, survey reports

**Table 3:** Notations used in this paper.

Notations	Descriptions
$u_{i}/v_{j}$	User/remote sensing resources
$I^{u_{i}}$	ID of a specific user
$v_{t_{i}}^{u_{i}}$	Interaction between the user and remote sensing resources
$A^{s}$	Similarity matrix among remote sensing resource categories
$A^{I}$	Adjacency relationship among remote sensing resource categories
{$v_{t_{1}}^{u_{i}},v_{t_{2}}^{u_{i}},v_{t_{3}}^{u_{i}},\ldots ,v_{t_{m}}^{u_{i}}$}	users’ historical behavior sequence

**Table 4:** Description of MovieLens, Amazon-clothes and Amazon-books datasets.

Datasets	MovieLens	Amazon-Clothes	Amazon-Books
Number of users	6040	4993	47,400
Number of remote sensing resources	3706	39	36,412
Data sparsity	95.53%	96.82%	99.99%

**Table 5:** Performance comparison on MovieLens, Amazon-clothes and Amazon-books.

Datasets	Models	k = 5			k = 10			k = 15			k = 20		
		Recall	Precision	F1	Recall	Precision	F1	Recall	Precision	F1	Recall	Precision	F1
MovieLens	CF	0.0755	0.0151	0.0252	0.1384	0.0138	0.0252	0.1824	0.0122	0.0228	0.2327	0.0116	0.0222
	NCF	0.0913	0.0182	0.0305	0.1633	0.0163	0.0297	0.2202	0.0147	0.0275	0.2593	0.0129	0.0247
	LSTM	0.0978	0.0196	0.0325	0.1889	0.0189	0.0343	0.2511	0.0167	0.0314	0.3578	0.0179	0.0341
	AGCN	0.1452	0.0290	0.0484	0.2253	0.0225	0.0410	0.2718	0.0181	0.0340	0.3195	0.0160	0.0304
	DCF	0.1523	0.0304	0.0508	0.2461	0.0246	0.0447	0.3041	0.0203	0.0380	0.3800	0.0190	0.0362
	**TGDL-RSSR**	**0.1565**	**0.0313**	**0.0522**	**0.2575**	**0.0257**	**0.0468**	**0.3165**	**0.0211**	**0.0396**	**0.4325**	**0.0216**	**0.0412**
Amazon-clothes	%Improv.	2.76%	2.96%	2.76%	4.63%	4.47%	4.70%	4.08%	3.94%	4.21%	13.82%	13.68%	13.81%
	CF	0.0494	0.0099	0.0165	0.1015	0.0102	0.0185	0.1439	0.0096	0.0180	0.2186	0.0109	0.0208
	NCF	0.0841	0.0168	0.0281	0.1362	0.0136	0.0248	0.1805	0.0120	0.0226	0.2423	0.0121	0.0231
	LSTM	0.0831	0.0166	0.0277	0.1592	0.0159	0.0289	0.2504	0.0167	0.0313	0.3444	0.0172	0.0328
	AGCN	0.1324	0.0221	0.0378	0.1809	0.0181	0.0329	0.2362	0.0157	0.0295	0.2987	0.0149	0.0284
	DCF	0.1061	0.0212	0.0354	0.1944	0.0194	0.0353	0.2748	0.0183	0.0343	0.3470	0.0173	0.0330
	**TGDL-RSSR**	**0.1465**	**0.0244**	**0.0418**	**0.2140**	**0.0214**	**0.0389**	**0.2925**	**0.0195**	**0.0366**	**0.3674**	**0.0184**	**0.0350**
Amazon-books	%Improv.	10.65%	10.41%	10.58%	10.08%	10.31%	10.20%	6.44%	6.56%	6.71%	5.88%	6.36%	6.06%
	CF	0.0744	0.0149	0.0248	0.1349	0.0135	0.0245	0.2186	0.0146	0.0273	0.2930	0.0147	0.0279
	NCF	0.0791	0.0158	0.0263	0.1823	0.0182	0.0332	0.2586	0.0172	0.0323	0.3805	0.0190	0.0362
	LSTM	0.1130	0.0226	0.0377	0.2300	0.0230	0.0418	0.3223	0.0215	0.0403	0.4093	0.0172	0.0390
	AGCN	0.1407	0.0281	0.0469	0.2488	0.0249	0.0452	0.3387	0.0226	0.0423	0.4243	0.0212	0.0404
	DCF	0.1354	0.0271	0.0452	0.2611	0.0261	0.0475	0.3678	0.0245	0.0460	0.4649	0.0233	0.0443
	**TGDL-RSSR**	**0.1487**	**0.0297**	**0.0496**	**0.2927**	**0.0293**	**0.0532**	**0.3933**	**0.0262**	**0.0492**	**0.4673**	**0.0234**	**0.0445**
	%Improv.	5.68%	5.69%	5.76%	12.10%	12.26%	12.00%	6.93%	6.94%	6.96%	5.16%	4.29%	4.51%

**Table 6:** Performance comparison on remote sensing service dataset.

Datasets	Models	k = 5			k = 10			k = 15			k = 20		
		Recall	Precision	F1	Recall	Precision	F1	Recall	Precision	F1	Recall	Precision	F1
Remote Sensing	CF	0.0733	0.0147	0.0244	0.1933	0.0193	0.0352	0.2600	0.0173	0.0325	0.3200	0.0160	0.0305
	NCF	0.0868	0.0174	0.0289	0.1837	0.0184	0.0334	0.2780	0.0185	0.0348	0.3744	0.0187	0.0357
	LSTM	0.1062	0.0212	0.0354	0.1948	0.0195	0.0354	0.2641	0.0176	0.0330	0.4245	0.0212	0.0404
	AGCN	0.1117	0.0224	0.0372	0.2001	0.0200	0.0364	0.2963	0.0198	0.0370	0.3966	0.0198	0.0378
	DCF	0.1293	0.0259	0.0431	0.2329	0.0233	0.0424	0.3365	0.0224	0.0421	0.4461	0.0223	0.0425
	**TGDL-RSSR**	**0.1279**	**0.0256**	**0.0426**	**0.2610**	**0.0261**	**0.0475**	**0.3576**	**0.0238**	**0.0447**	**0.5190**	**0.0260**	**0.0494**
	%Improv.	−1.08%	−1.16%	−1.16%	12.07%	12.02%	12.03%	06.27%	06.25%	06.18%	16.34%	16.59%	16.24%

**Table 7:** Comparison of remote sensing resource retrieval methods.

Comparison Items	Active Service Recommendation Model	Content-Based Retrieval Method
Service mode	Proactive	Passive
User satisfaction	2	0
System usage time	4.5 min	8.3 min
System latency time	0.58 s	1.13 s

## Data Availability

Data are contained within the article.
